# Temperature Distribution and Vertical Stratification near Side-Wall Inlets of Stacked Cage Broiler Houses in Winter

**DOI:** 10.3390/vetsci13101103

**Published:** 2026-10-16

**Authors:** Yongxiang Wei, Wenjia Li, Quanyou Sun, Shaoyu Li, Bin Xu

**Affiliations:** 1 Institute of Animal Husbandry, Henan Academy of Agricultural Sciences, Zhengzhou 450002, China; weiyongxiang2025@163.com (Y.W.);; 2 Henan Key Laboratory of Animal Breeding and Nutritional Regulation, Zhengzhou 450002, China

**Keywords:** broiler house, side-wall inlet, temperature distribution, vertical stratification, ventilation, thermal environment, animal welfare, thermal comfort

## Abstract

Inlet design assumes that supply air from side-wall inlets mixes with house air over several metres, but whether this holds in a densely caged multi-tier house, where cages and birds obstruct the jet, is untested. The bird-zone environment of a commercial stacked-cage house was measured with 24 in-cage sensors at three distances and two tiers over ten winter days. Three of four groups showed no resolvable cooling gradient at 1.5 m; only one north-side group cooled with distance, strengthening with ventilation (0.30 K per percent, R^2^ = 0.39), while another showed the opposite sign. Every fitted decay coefficient ($\alpha d_{h}=0$ to 0.004) lay below the free-jet design range (0.01–0.03), by five or more times over the window. Vertical stratification of 0.78 K dominated (side × tier p<0.001): the upper tier was up to 2.4 K warmer on the north side, while the south-side tiers differed by under 0.5 K. Shading nets lowered north-side inlet temperature by 0.80 K but warmed the south side. The practical consequence is that the thermal environment is organised by tier and side rather than by near-inlet distance, so monitoring, control and welfare-oriented assessment should target tiers and sides.

## Simple Summary

In winter, broiler houses run at the minimum ventilation that keeps air quality acceptable, and the cold incoming air is expected to mix with the room air on its way to the birds. The air temperature inside the cages of a commercial four-tier broiler house was measured for ten days and compared with the decay pattern that inlet design assumes. Three of the four inlet groups showed no resolvable decay over the analysis window; the largest coefficient, in the group nearest the exhaust fans under high ventilation ($\alpha d_{h}$ = 0.004), lay below the design range (0.01–0.03), as did every other fit. The differences birds experienced were dominated by cage tier and side: the upper tier was 0.8 K warmer than the lower one, up to 2.4 K on the north side, while the south-side tiers differed by less than 0.5 K. Shading nets lowered the north-side inlet temperature by 0.8 K but raised the south side, so their benefit depends on the side of the house. The practical implication is that the thermal environment is organised by tier and side rather than by the near-inlet distance, so monitoring and adjustment should target tiers and sides.

## 1. Introduction

The broiler breeding industry is a vital component of animal husbandry, and in China, production has increasingly shifted toward stacked-cage rearing systems that offer large feeding capacities and high space utilization. Within mechanically ventilated houses, side-wall inlet jets are the primary mechanism for distributing fresh air. The design of these inlets directly affects bird welfare, energy consumption, and production efficiency, as temperature and stocking density jointly affect physiological function and growth performance [1,2]. Well-designed inlets create jets that travel across the ceiling, entrain warm air from the upper zone, and deliver a mixed airstream to the bird-occupied zone [3]. In cold-climate poultry production, minimizing heat loss through ventilation while maintaining air quality is a key design challenge, and dynamic thermal load simulation provides a basis for optimizing insulation and heating requirements [4].

The practical problem is sharpest in winter, when the controller runs at minimum ventilation and the supply air is at or below freezing. That air enters directly into the occupied zone through side-wall inlets, so how far it travels before reaching thermal equilibrium sets two things at once: the temperature the birds actually experience, and the ventilation heat load. Inlet sizing and staging are therefore based on a predicted jet throw, and the throw is estimated from the free-jet temperature-decay coefficient α, whose dimensionless form $\alpha \cdot d_{h}$ is taken as 0.01–0.03 for slot inlets [5,6]. Those coefficients come from unobstructed jets in empty or lightly loaded enclosures. In a commercial broiler house the jet passes through a dense array of cages, birds and equipment that is expected to shorten its throw considerably, so the design correlations may not describe the first metres that matter most for the birds–yet field measurements inside the occupied zone of a multi-tier cage house are scarce. Sensor-based monitoring and early-warning systems for caged broiler houses are being developed to capture exactly this spatial heterogeneity [7], and short-term thermal forecasting is being explored to feed predictive control [8,9]; both would benefit from a measured picture of where the bird-level differences actually sit.

Winter temperature stratification in broiler houses has been documented across multiple production systems. Wheeler et al. [10] measured vertical differences of 1–3 K between floor and ceiling in Pennsylvania houses, noting that stratification intensified with building height and weakened with higher ventilation. At bird level, Boshouwers et al. [11] reported differences of 0.5–2.0 K depending on the balance between heat production, ventilation rate, and building geometry. More recent CFD studies have confirmed the importance of building geometry and microclimate conditions on these gradients [12]. Under minimum winter ventilation, Xin et al. [13] found that vertical gradients became more pronounced in broiler houses using either conventional side-wall ventilation or tunnel ventilation, and Cayli [14] further quantified spatial variability of temperature and humidity in tunnel-ventilated broiler houses. Data-driven analyses of multi-sensor records in winter broiler houses likewise highlight spatial and temporal structure in the environmental field [15]. More recently, Zheng et al. [16] observed similar side-dependent stratification in cage layer barns, attributing it to heat accumulation in upper spaces, while Babadi et al. [17] reported coupled distributions of temperature, CO_2_, and NH_3_ in caged layer houses.

Jet temperature decay in livestock buildings is typically modeled as $T\left(x\right)=T_{0}\cdot \exp\left(-\alpha \cdot x\right)$, where α is the decay coefficient [5]. The value of α depends on inlet geometry, velocity, and the temperature differential between jet and ambient air [6]. Zhang et al. [18] developed jet-drop models for non-isothermal, flap-adjusted ventilation jets, and later extended this work to multi-inlet systems [19]. Inlet performance standards established by Hoff and Wu [20], together with work by Oberreuter and Hoff [21] on static pressure and opening angle, provide a framework for evaluating commercial inlet designs. At the dimensionless level, Adre and Albright [22] proposed similarity criteria for slotted-inlet enclosures, and Leonard and McQuitty [23] showed that buoyancy becomes significant when the Archimedes number exceeds 0.01. Kwon et al. [24] used CFD to demonstrate that building geometry and internal obstructions strongly influence jet behavior in broiler houses. Blanes-Vidal et al. [25] validated CFD predictions of airflow patterns in mechanically ventilated poultry buildings, confirming that accurate representation of inlet boundary conditions is essential for reliable simulations. More recent CFD studies have extended this approach to simulate dynamic temperature variations under tunnel ventilation [26] and to model three-dimensional temperature distributions across the entire broiler house volume [27]. Ma et al. [28] also quantified the influence of tunnel ventilation on the indoor thermal environment of poultry buildings in winter, demonstrating that ventilation strategy directly shapes the temperature field at the house level.

Most prior work has addressed free or weakly confined jets. Commercial stacked-cage houses present a fundamentally different environment: multiple cage tiers, high bird density, and equipment create complex flow patterns that cannot be predicted from free-jet theory alone. The same obstruction problem applies wherever birds and equipment occupy the ventilated volume, so the question also bears on how broadly free-jet design rules can be transferred between housing systems. The consequence is a specific, testable gap: it is not known whether the design decay coefficients ($\alpha \cdot d_{h}=0.01$–0.03) describe the temperature field that the birds actually occupy in a multi-tier cage house, or whether the cage array mixes the supply air so quickly that the design correlations are inapplicable over the distances that matter. A previous study at the same farm assessed temperature distribution at the house level, documenting lateral differences driven by solar radiation and wind [29]. That study focused on horizontal gradients and did not resolve near-inlet decay profiles or vertical stratification between tiers—two factors critical to understanding how fresh air reaches birds at different heights. Building on that house-level baseline, the present study zooms into the first 10 m from the inlet wall to resolve these near-field details.

Beyond the jet itself, the inlet boundary condition also shapes the near-field temperature: shading nets modify local wind speed and radiative gain at the inlet plane and thus reset the thermal baseline on which the decay profile develops. Shading nets are routinely installed outside side-wall inlets in cold regions to deflect wind and reduce heat loss, yet their effect on specific inlet groups has not been quantified with paired measurements. Together, the decay profile, its ventilation dependence, the inter-tier stratification, the dimensionless flow regime, the spatiotemporal field and the shading-net effect provide a complete near-inlet picture for zone-specific ventilation design, i.e., for targeting monitoring and control at the tiers and the two sides rather than at the near-inlet zone alone.

Thermal conditions are among the most consequential environmental inputs to broiler welfare. Broilers thermoregulate within a comfort band that narrows as they grow, and cold stress, heat stress and the inability to perform comfort behaviour are recognised welfare consequences in commercial broiler production [30]; welfare assessment in practice therefore combines environmental records with animal-based indicators [31]. Continuous, automated monitoring of the animal environment is the direction precision livestock farming takes for welfare assessment and management [32,33]. Because the thermal environment of a commercial house is not uniform, the resolution at which it is measured and controlled is itself a welfare question: a single house-average set-point can leave birds in one part of the house outside the intended band while the controller reports compliance. The present measurements are taken where the birds actually sit, at two cage tiers, three distances and both sides of the house, and therefore describe the thermal conditions that welfare assessment would need to resolve.

This field study had six objectives: (1) quantify the near-inlet temperature gradient using an exponential model with non-negative constraint; (2) evaluate how ventilation rate modifies the gradient; (3) characterize vertical stratification between cage tiers; (4) assess the consistency of observed patterns with momentum-driven jet behavior through dimensionless analysis; (5) characterize the spatiotemporal temperature field and its coupling to outdoor conditions; and (6) measure shading-net effects via paired comparison. The result is a directly measured comparison between the near-inlet thermal field of a commercial multi-tier cage house and the free-jet decay coefficients used to design such houses.

## 2. Materials and Methods

### 2.1. Experimental Facility

**Figure 1:**
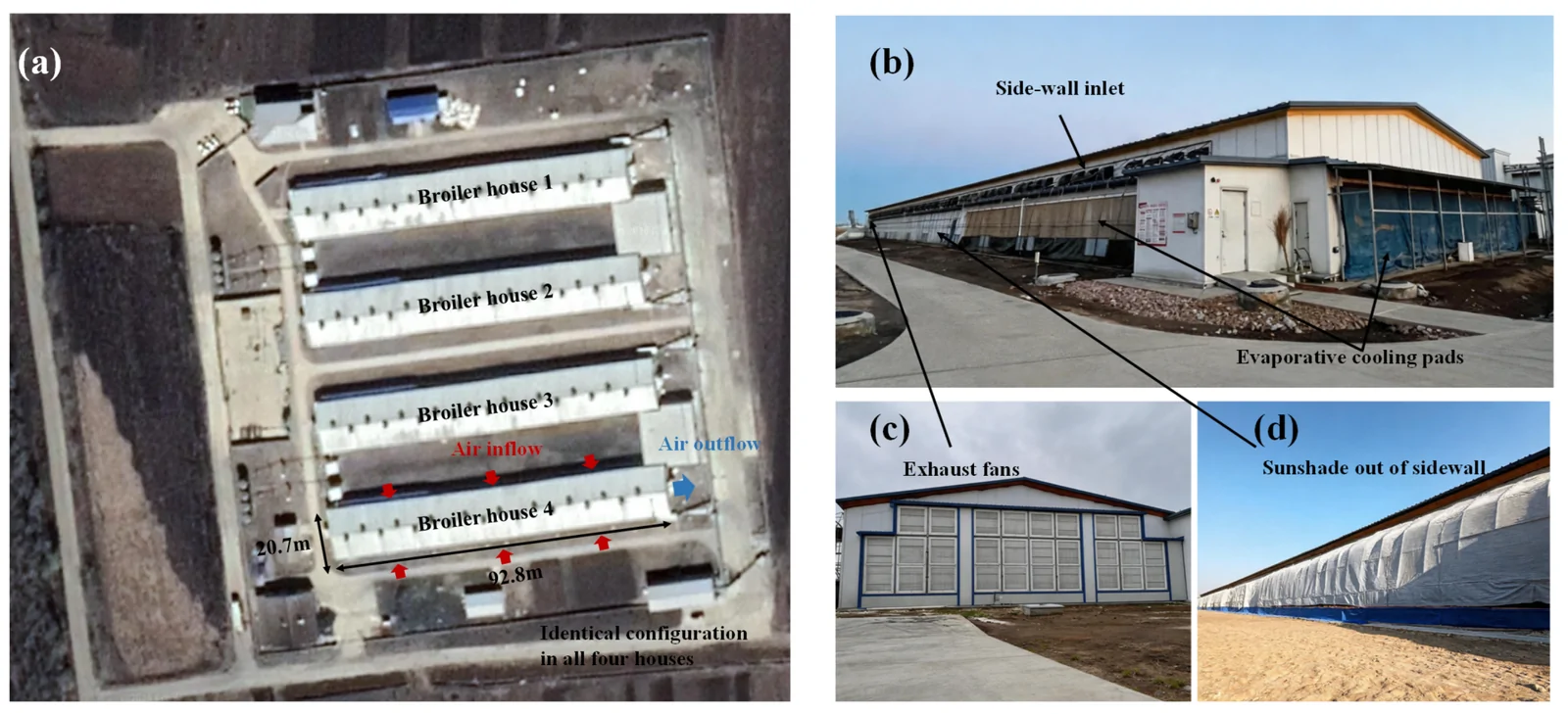
Location and layout of the commercial broiler farm (Jilin Province, China). (**a**) Satellite view of the four houses, with Building 4 highlighted; (**b**) side-wall inlets with the evaporative cooling pads above them; (**c**) exhaust fans on the end wall; (**d**) sunshade net over the sidewall. The cooling pads were not operated during the winter study.

The experiment was conducted at a commercial broiler farm in Jilin Province, China (44°50′ N, 126°32′ E) during February–March 2023. The farm comprised four identical houses, each oriented east–west and measuring 92.8 m in length, 20.7 m in width, and 6.5 m in height. Each house held approximately 78,000 broilers (Arbor Acres) in a stacked-cage arrangement with six rows and four tiers (cage dimensions: 2.4 m × 1.8 m × 0.65 m per cage, 95 broilers per cage). The study focused on Building 4 (Figure 1). The monitored flock was a single batch of day-old chicks reared to slaughter in place over 38 d; the measurement window covered the final stage of this batch (days 28–38), and procedures outside that window, including those between production cycles, were not part of the measurement campaign.

The building envelope was constructed from foamed polyurethane panels with 100-mm-thick walls (heat-transfer coefficient 0.18 W m^−2^ K^−1^) and a 120-mm-thick roof (heat-transfer coefficient 0.21 W m^−2^ K^−1^). Each sidewall was equipped with 50 side-wall inlets, each measuring 0.82 m × 0.33 m and positioned 3.4 m above the floor. At the opposite end wall, 24 exhaust fans were installed: 22 fans (EM52, Munters, Kista, Stocksund, Sweden; 1.42 m diameter, 40,000 m^3^ h^−1^ each) and 2 smaller fans (EM36, Munters, Kista, Stocksund, Sweden; 1.1 m diameter, 20,000 m^3^ h^−1^ each). Eight air–water heat exchangers (Heat-X Type-4H, Reventa GmbH, Kista, Stocksund, Sweden; 7500 m^3^ h^−1^, 95 kW each) supplied by a natural gas boiler (WNS2.8, Harbin Hongguang Boiler Group Co., Ltd., Harbin, China; 2.8 MW) maintained the indoor temperature during cold weather.

In winter, the controller modulated fan operation based on indoor temperature and CO_2_ concentration, running in minimum-ventilation mode with intermittent fan operation; its ventilation output is recorded as a commanded position (%; Section 2.3). Throughout the study period all ventilation air entered through the side-wall inlets: the evaporative cooling pads and the longitudinal (tunnel) inlets at the pad end wall stayed closed (longitudinal-window opening held at about 1%), so the side-wall inlets were the only supply path. The design minimum ventilation rate is indexed to bird age and rises from 3280 to 60,020 m^3^ h^−1^ over the production cycle; across the analysis window the controller’s temperature setpoint declined from 24.3 to 20.9 °C, tracking the growing flock’s declining thermal requirement. Daily body-weight and feed-intake records for this batch were not available; stage-level performance for the same house and winter is reported by Deng et al. [29]. A 6-needle sunshade net was installed over both sidewalls to reduce wind effects on inlet ventilation. Manure was removed by belt conveyors. Feed was delivered three times daily via two round feed pans per cage, with ad libitum access between deliveries; each cage was equipped with nipple drinkers (14 per cage). The farm lighting programme used full daytime illumination without dimming on days 1–3 and 32–38 (lights off for 1–2 h from 23:00), and dimmed lighting from 11:00 with an evening dark period of 19:00–21:00 on days 4–31 (see Section 4.6).

### 2.2. Temperature Measurements

**Figure 2:**
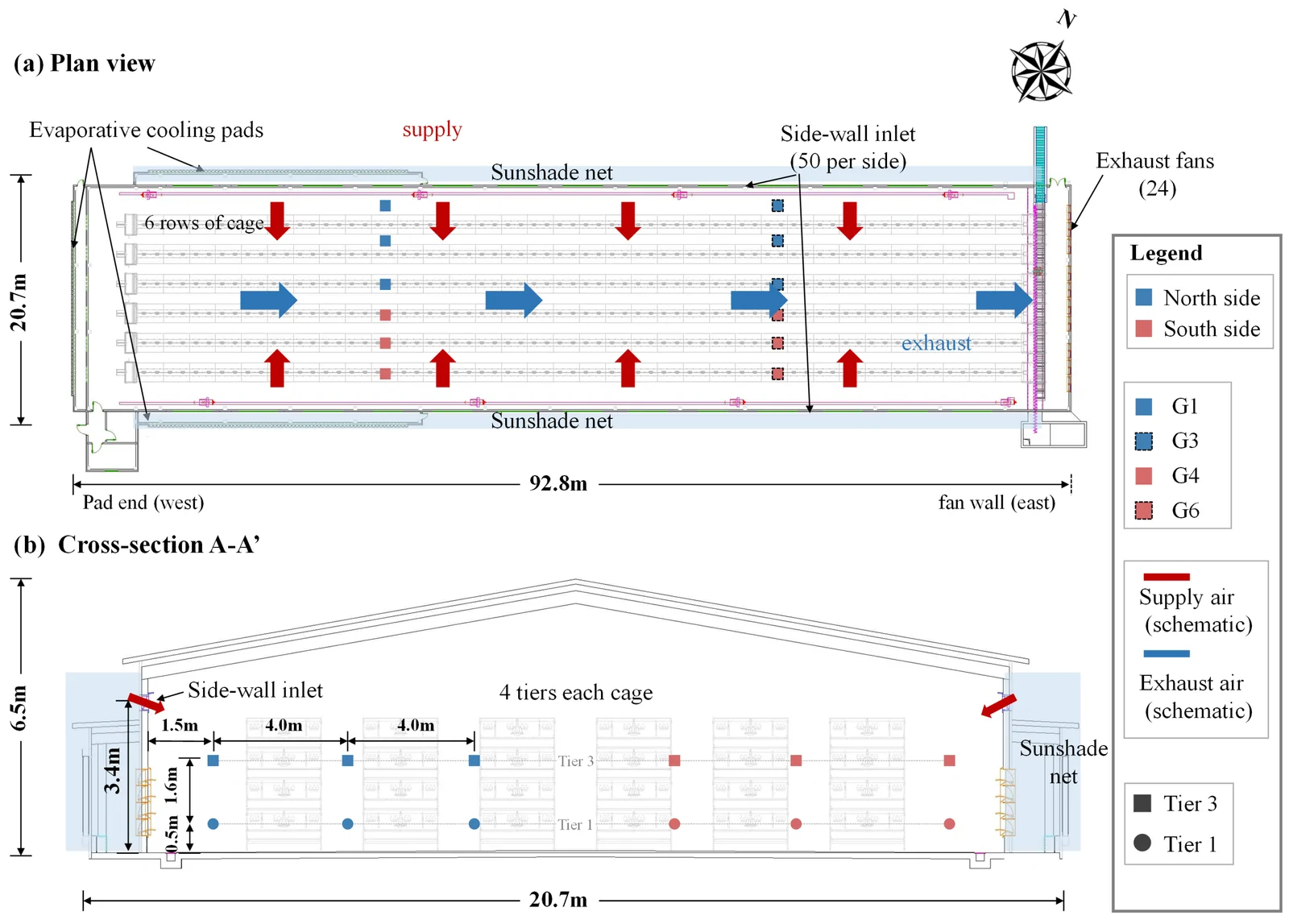
Distribution of the 24 temperature sensors in Building 4. Plan view (**a**) and cross-section (**b**). Sensors were arranged in four groups—G1 and G3 on the north sidewall (blue), G4 and G6 on the south sidewall (red)—each with three measurement positions at 1.5, 5.5, and 9.5 m from the inlet wall and two vertical tiers. In the plan view each mark denotes one measurement position, without distinguishing tiers; solid outlines = G1/G4 (pad end) and dashed = G3/G6 (fan wall). In the cross-section, squares = Tier 3 (2.1 m) and circles = Tier 1 (0.5 m); arrows indicate the winter airflow scheme (red = supply, blue = exhaust) and are schematic.

Twenty-four temperature loggers (HOBO Pro v2, accuracy ±0.2 K) were installed inside the cages of Building 4 to sample the bird-occupied zone at increasing distance from the side-wall inlets. Sensors were arranged in four of the farm’s inlet groups (Groups 1 and 3 on the north sidewall; Groups 4 and 6 at the analogous longitudinal positions on the south sidewall; the remaining groups were not instrumented because the number of loggers was limited). Group 1 lies near the pad end of the house and Group 3 near the exhaust-fan wall, with Groups 4 and 6 at analogous longitudinal positions on the south sidewall. Within each group, sensors were placed at three measurement positions at increasing distance from the sidewall (1.5, 5.5 and 9.5 m; the middle field of the sensor identifiers (7, 17 and 27) denotes these three positions) and at two vertical heights, 0.5 m and 2.1 m above the floor (Tier 1 and Tier 3, following the sensor identifiers; see cross-section in Figure 2). Tier 1 samples the lower bird zone and Tier 3 the upper bird zone, bracketing the vertical gradient; the remaining cage tiers were not instrumented (see Section 4.6). The 24 sensors therefore provide 12 horizontal positions × 2 tiers. The sensors sample the bird-occupied zone rather than the free space above the cages, and the paired vertical measurements separate the jet-related and stratification-related contributions to bird-level temperature.

Data were recorded at 1-min intervals from 14 to 24 March 2023, corresponding to the fourth fattening stage (bird age 28–38 days) under winter minimum ventilation. Outdoor air temperature during this window ranged from −10.1 to 16.3°C (median 0.4°C, with 46% of records below 0°C), and the heating circuit was closed for most of the period but still switched on intermittently (about 13% of controller records). All records were screened with a physical-range filter (−10 to 50°C) before any analysis, and sensor IDs follow the group–cage–tier convention (e.g., 1-27-1 denotes Group 1, cage position 27, Tier 1). Four sensors required treatment in the 14–24 March record. Two north-side sensors at the 9.5 m position (1-27-1 and 3-27-1, both Tier 1) recorded no valid data and were excluded from all analyses. Sensor 4-17-1 was degraded (72% of its records outside the valid range or missing) and contributed only its valid records. Sensor 6-17-3 failed intermittently (readings from −95 to −62°C with sporadic recoveries that did not track its neighbouring sensor) and was excluded from the spatiotemporal heatmap (Section 3.5), while the few physically plausible records it produced were retained in the group statistics. All remaining sensors had <1% missing values. Statistics are therefore computed on the valid records available for each sensor, and Section 3.3 reports a sensitivity analysis in which both degraded sensors are removed entirely. The indoor 24-sensor record (17 February–24 March) shows additional degraded channels outside the analysis window but does not affect the 14–24 March results.

### 2.3. Outdoor and Ventilation Data

Outdoor temperature, humidity, wind speed, and wind direction were recorded at 15-min intervals from an on-farm weather station (Hobo Pro Series, Onset Computer Corp., Bourne, MA, USA). Because the station carried no solar-radiation sensor, hourly shortwave radiation for the site was taken from the ERA5 reanalysis [34] served by the Open-Meteo historical-weather archive [35]. Static pressure was recorded on both sidewalls with differential pressure transducers (accuracy ±1 Pa), but only the north-side records were usable for the analysis. These come from four inlet positions along the house (positions 1, 5, 9 and 13 of the farm’s inlet numbering, i.e., bays 1, 5, 9 and 13 of the 13 bays along the building; referred to here as P1, P5, P9, P13 and separate from the temperature groups G1/G3/G4/G6).

Ventilation data—fan status, ventilation position (%), window openings, and CO_2_ concentration—were obtained from the house climate controller at 10-min intervals. The CO_2_ channel is the controller’s own building-level sensor (mean 3022 ppm, range 2062–3854 ppm during 14–24 March); no independent calibration was performed in this campaign, so it is used here only as building-level context. The controller’s ventilation position (%), used as the ventilation-rate variable in Section 3.1, Section 3.2, Section 3.3, Section 3.4 and Section 3.5, is a commanded actuator setting that scales fan and inlet operation within the controller’s configured range; it is not a measured volumetric flow, and the side-window opening (%) and longitudinal-window opening (%) are separate actuator records. Shading net status (open/closed) was recorded for both sides of Building 4, with paired sensors installed both outside the net (exposed inlet) and inside the net (under the shade) to measure the thermal effect directly; these paired records were logged at 5-min intervals over 7–13 March 2023 (Section 3.6), a period separate from the 14–24 March core analysis window. Wind direction data were converted to the vector component perpendicular to the sidewall for correlation analysis.

### 2.4. Mathematical Framework

The mathematical models are consolidated below.

**Exponential decay.** Temperature decay along the jet path was modeled as

(1) $$T\left(x\right)=T_{0}\cdot \exp\left(-\alpha \cdot x\right)$$

where *x* is distance from the inlet wall (m), $T_{0}$ is the inlet temperature (°C), and α is the decay coefficient ($m^{-1}$). The model was fitted via non-linear least squares (curve_fit, SciPy) with the constraint α≥0; negative α would imply warming with distance, which is physically implausible for a cold jet entering a warm house. Bootstrap resampling (1000 iterations) provided 95% confidence intervals for α. Alternative forms—power law $T\left(x\right)=T_{0}\cdot x^{-b}$ and linear $T\left(x\right)=T_{0}-\beta \cdot x$—were compared using AIC and BIC.

**Physical attenuation and inlet velocity.** From Bernoulli and energy balance, the inlet air speed relates to the static pressure drop ΔP (Pa) as

(2) $$v_{\mathrm{inlet}}=\sqrt{2\Delta P/\rho }$$

where ρ=1.2 kg m^−3^ is the air density. For momentum-dominated jets, the decay coefficient scales inversely with inlet velocity:

(3) $$\alpha \propto 1/v_{\mathrm{inlet}}$$

Higher-velocity jets carry greater momentum, penetrate further, and mix more slowly with ambient air [3]. The model was evaluated with pressure data from the four north-side pressure positions (P1, P5, P9, P13; south-side verification was not possible, see Section 4.6). Ventilation rate was analyzed both as a categorical variable (tertiles, Section 2.5) and as a continuous variable (Section 2.5).

**Dimensionless parameters.** Flow regime and buoyancy were characterized by the Reynolds number and Archimedes number, respectively:

(4) Re=vinlet·dh/ν

(5) $$\mathrm{Ar}=g\cdot \beta \cdot \Delta T\cdot d_{h}/v_{\mathrm{inlet}}^{2}$$

where $d_{h}=0.47$ m is the hydraulic diameter of the 0.82×0.33 m slot inlet (2ab/(a+b)), $\nu =1.5\times 10^{-5}$ m^2^ s^−1^ is the kinematic viscosity (distinct from $v_{\mathrm{inlet}}$, the inlet speed), g=9.81 m s^−2^, β=1/300 K^−1^ is the thermal expansion coefficient, and ΔT=5 K is the representative winter jet-ambient temperature difference (in kelvin; see Section 2.6). Leonard and McQuitty [23] showed that buoyancy becomes significant when Ar>0.01. In this study, Re(t) and Ar(t) were summarized as median and percentile statistics and as time-varying distributions from the 1-min static-pressure series (Section 3.4), and compared with the literature range $\alpha \cdot d_{h}\approx 0.01$–0.03 for free slot jets [5,6].

**Model comparison.** Goodness-of-fit for the exponential, power-law, and linear models was assessed via

(6) $$\mathrm{AIC}=2k-2\ln(\hat{L})$$

(7) $$\mathrm{BIC}=k\ln\left(n\right)-2\ln\left(\hat{L}\right)$$

where *k* is the number of parameters and $\hat{L}$ the maximized likelihood; $R^{2}=1-{\mathrm{SS}}_{\mathrm{res}}/{\mathrm{SS}}_{\mathrm{tot}}$ is reported for completeness. When no decay curvature is present the exponential model cannot be fitted, so AIC and BIC are undefined.

### 2.5. Ventilation and Outdoor Coupling Analysis

Ventilation data—fan status, ventilation position (%), CO_2_ concentration (building level only), and negative pressure—were obtained from the house controller at 10-min intervals (merged to the 1-min cage temperature series with a 10-min nearest-neighbour tolerance, yielding 100% match). Ventilation position (hereafter ventilation rate, %) was analyzed both categorically (tertiles: Low < 7.0%, Mid 7.0–9.3%, High > 9.3%, with cut points derived from the raw ventilation distribution to avoid duplication bias; see Section 3.2) and continuously via ordinary least squares regression $\Delta T=\beta_{0}+\beta_{1}\cdot \mathrm{vent}\%$ (Section 3.2). The continuous regression is univariate by design: ventilation rate was the single predictor, fitted separately for each inlet group. Indoor humidity, negative pressure, and CO_2_ were recorded by the controller but were not entered as covariates: they are strongly collinear with the ventilation rate (Spearman |ρ| up to 0.85) because they are jointly determined by the same control action, so they cannot separate its effect from their own, and CO_2_ exists only at building level. Section 3.2 reports a sensitivity analysis that adds them individually.

Outdoor temperature, humidity, wind speed, and wind direction were recorded at 15-min intervals from the on-farm weather station. Wind direction was converted to the component perpendicular to the sidewall. Outdoor conditions were paired to the hourly ΔT series via a 30-min tolerance and analyzed as boundary conditions (Spearman correlations, day/night stratification; Section 3.5).

### 2.6. Statistical Analysis and Units

One-way analysis of variance (ANOVA) with Tukey’s honestly significant difference (HSD) post-hoc tests compared mean temperatures across inlet groups. Two-way ANOVA (Type II, Section 3.3) tested the effects of side (north/south), tier (lower/upper), and their interaction, with full reporting of SS, df, MS, F, p, $\eta^{2}$, and partial $\eta_{p}^{2}$. Assumptions were checked via Shapiro-Wilk (residuals) and Levene’s (cell) tests; a linear mixed-effects model with a random intercept for sensor was fitted as a sensitivity analysis accounting for repeated measures over time (Section 3.3), and the intraclass correlation coefficient was computed from its variance components as the ratio of the sensor-level variance to the total (sensor plus residual) variance, on a random subsample of 80,000 observations. Shading-net effects were evaluated with paired *t*-tests; Cohen’s d quantified effect size. Hourly ΔT series were correlated with outdoor temperature, wind speed, ventilation rate, and reanalysis shortwave radiation using Spearman’s rank correlation, which measures monotonic association and is robust to the skewed, outlier-bearing distributions of these series; day (08:00–16:00) and night sub-periods were compared with Mann-Whitney U tests (Section 3.5).

The spatiotemporal heatmaps (Section 3.5) show hourly means of the 1-min records on the distance × time grid of each group (tiers pooled; distances 1.5, 5.5 and 9.5 m), displayed with nearest-neighbour cells and no spatial or temporal interpolation; hours without valid records are shown in grey. All statistics use the valid records defined in Section 2.2, and Section 3.3 reports the sensitivity analysis excluding the two degraded sensors. The longitudinal position relative to the exhaust fans was accounted for by fitting each inlet group separately (G3/G6 proximity; Section 3.2). Temperature differences (ΔT) are reported in kelvin (K) throughout, while absolute temperatures remain in °C. Vertical differences are computed in two ways, and the distinction is stated where each is used: pooling all valid records (the pooled group-level values and the sensitivity analysis in Section 3.3) or pairing each tier’s available distances in time (the day-night and split-window comparisons in Section 3.5 and Section 4.5). The total installed fan capacity is 920,000 m^3^ h^−1^ (22 × EM52 at 40,000 m^3^ h^−1^ + 2 × EM36 at 20,000 m^3^ h^−1^), and the controller realised the age-indexed minimum-ventilation schedule (Section 2.1) by modulating fan operation.

## 3. Results

### 3.1. Temperature Decay and Model Comparison

The near-inlet temperature field was tested for a measurable gradient over 1.5–9.5 m and for its dependence on the ventilation rate.

**Figure 3:**
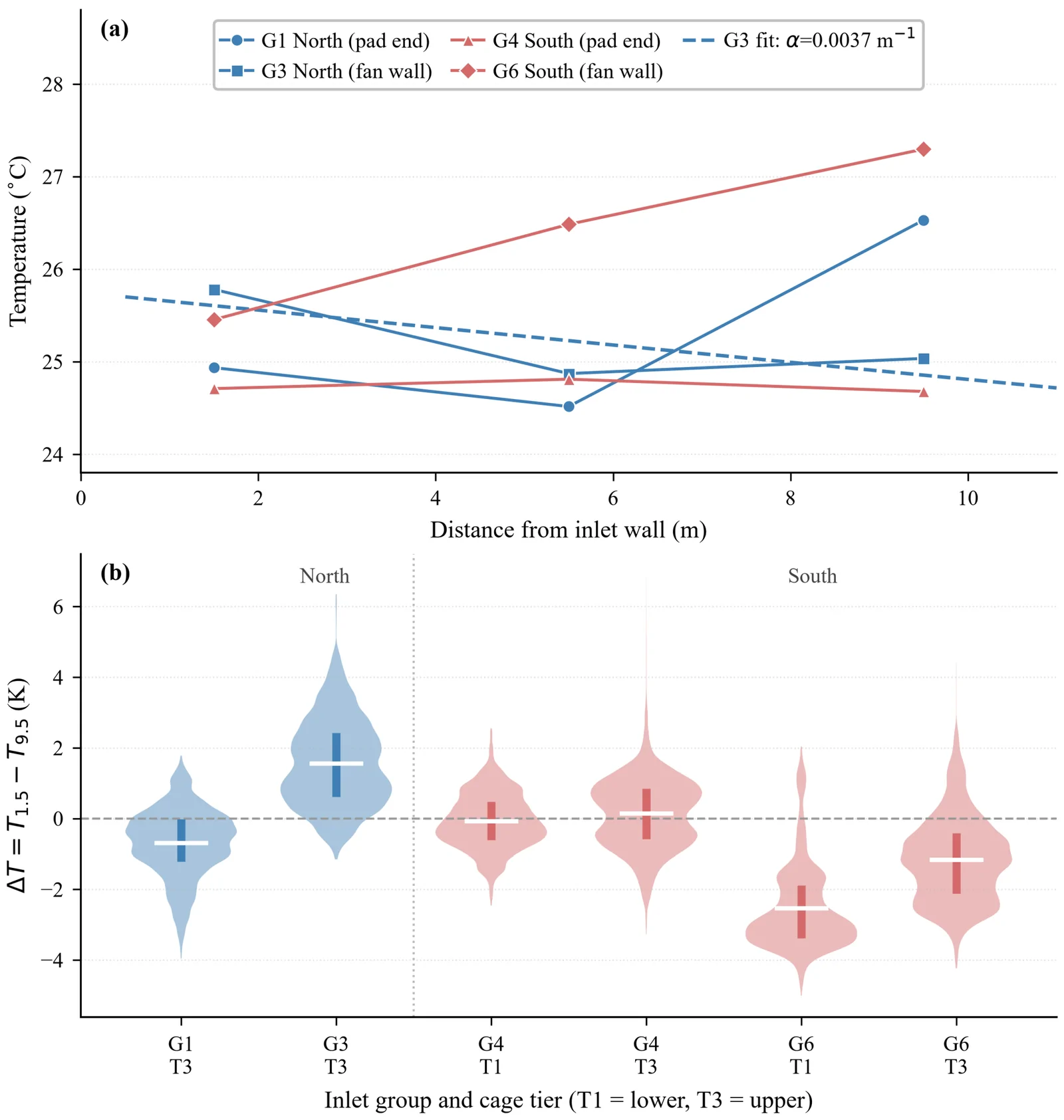
(**a**) Mean temperature against distance from the inlet wall; the dashed line is the fitted exponential decay for Group 3, the only group with a resolvable fit (its 95% bootstrap band is narrower than the line width). (**b**) Violin plots of $\Delta T=T_{1.5m}-T_{9.5m}$ by group and tier; white bars = means, coloured bars = interquartile range. Tier 1 is absent for G1 and G3 because two sensors were excluded (Section 2.2), so the north-side Tier 1 statistics rest on the 1.5 and 5.5 m sensors only (Section 3.3).

The exponential decay model (Equation (1)) was fitted to three spatial points per group (at 1.5, 5.5, and 9.5 m from the inlet wall). Three of four inlet groups showed negligible decay coefficients (α≈0 for Groups 1, 4, and 6, Figure 3a; Table 1), with standard errors comparable to or exceeding the estimates themselves. Only Group 3 (north side) had a measurable coefficient (α = 0.0037 m^−1^, R^2^ = 0.60); the measured mean at each instrumented position is given in Table 2.

To complement the three-point model, the full time series was used to compute the temperature difference $\Delta T=T_{1.5m}-T_{9.5m}$ at each paired time point (n>14,000 per group per tier). Group 3 showed a clear positive ΔT (mean 1.56 K, t=150.19, d=1.26, p<0.001; Figure 3b), confirming measurable cooling with distance. Group 4 showed a mean ΔT near zero (Tier 3: 0.14 K, d=0.14; Tier 1: −0.08 K, d=−0.10), indicating that the jet reached near-equilibrium conditions before the first measurement point at 1.5 m. Groups 1 and 6 both showed negative mean ΔT, but of very different magnitude: Group 1 Tier 3 averaged −0.69 K (d=−0.71) whereas Group 6 averaged −2.53 K at Tier 1 (d=−2.09) and −1.15 K at Tier 3 (d=−0.96). A negative ΔT means the air 9.5 m from the sidewall was warmer than the air at 1.5 m, i.e., the opposite sign to the near-inlet cooling a cold supply jet would produce; Group 6 therefore does not support the jet-decay interpretation at all, and its behaviour is discussed in Section 4.1.

For Group 3, the power law gave the lowest AIC (Equation (6)) among the three candidate forms (AIC = −6.46, R^2^ = 0.80), followed by the exponential (AIC = −4.28, R^2^ = 0.60); with only three spatial points per group the comparison is descriptive rather than discriminative, and neither form fits the three points well. The exponential is nevertheless the form whose coefficient design practice uses, so it is retained for the comparison with the design range—with the caveat that its three-point fit under-states the domain change: it predicts 0.75 K between 1.5 and 9.5 m where the full time series gives 1.56 K (Tier 3). For groups with α≈0, the exponential fit was numerically degenerate because no decay curvature was present, so a three-form comparison is not meaningful for those groups. The three-point fit captures the net change across the domain, not the shape of the profile within it: for Group 3 the fitted parameters ($T_{0}=25.75\;{}^{\circ}$C, α=0.0037 m^−1^) predict a difference of $T_{0}\left[\exp\left(-0.0037\times 1.5\right)-\exp\left(-0.0037\times 9.5\right)\right]=0.75$ K between 1.5 and 9.5 m, whereas the full time series gives 1.56 K (Tier 3), so the aggregate change is larger than the three-point summary suggests.

### 3.2. Ventilation Dependence of the Gradient

If higher inlet velocity produces a longer penetration zone, then the temperature gradient should increase with ventilation rate. This hypothesis was tested by stratifying the data by ventilation tertile.

**Figure 4:**
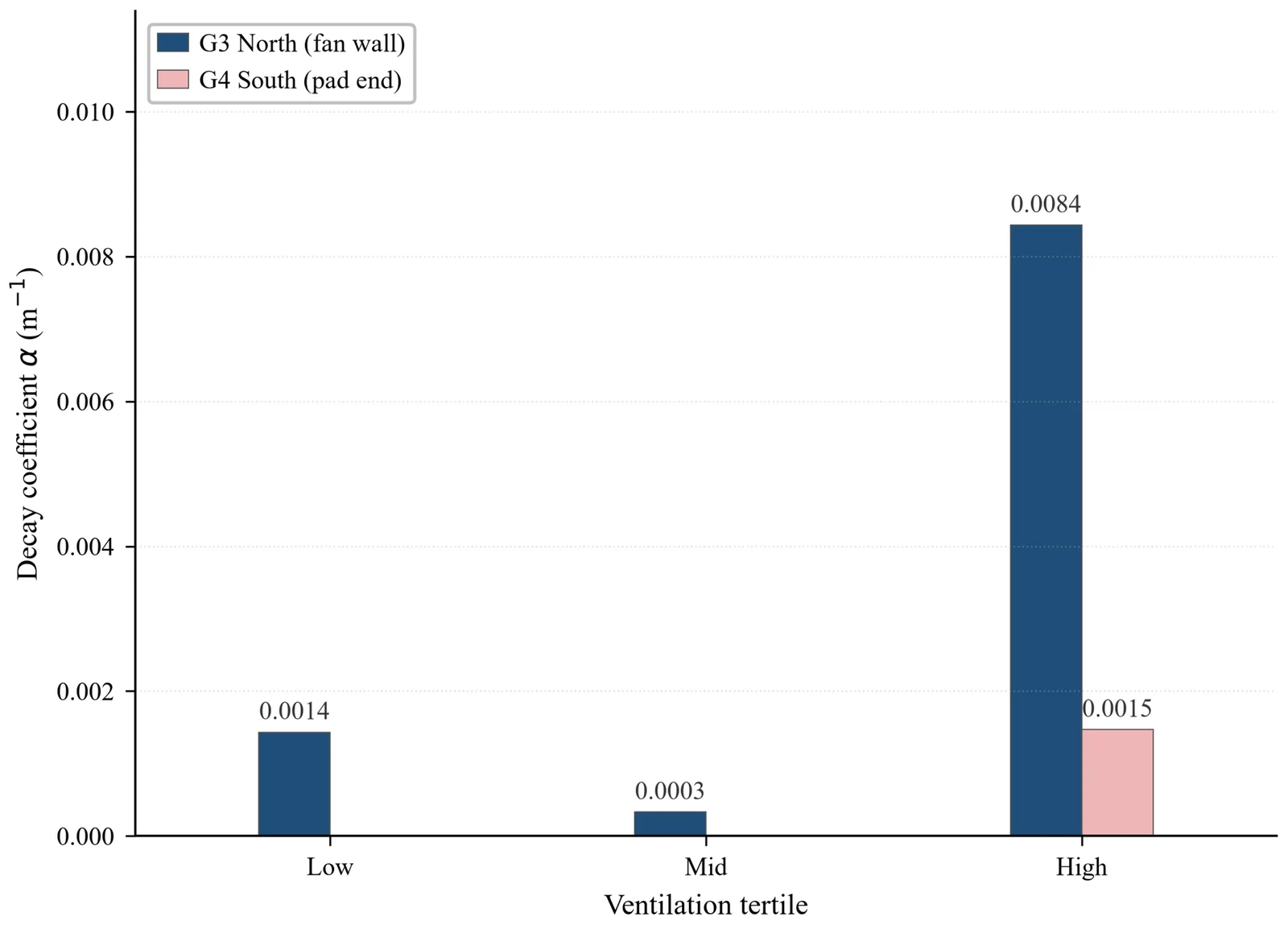
Decay coefficients stratified by ventilation tertile. Bars are shown for the two groups with a resolvable coefficient (G3, north, fan wall; G4, south, pad end) and are labelled with the fitted value; Groups 1 and 6 were zero to numerical precision at every tertile and are not shown. Tertiles are bins of the controller’s ventilation record (Section 2.5); the spatial positions of the groups are shown in Figure 2.

Stratifying by ventilation tertile revealed a clear pattern (Figure 4). Group 3 was the only group with a resolvable decay coefficient in all three bins (α = 0.0014, 0.0003 and 0.0084 m^−1^ for low, mid and high ventilation), and Group 4 showed weak decay under high ventilation (α = 0.0015 m^−1^); Groups 1 and 6 remained numerically zero at all three levels. Decay is therefore not uniformly absent, but it is pronounced only in the group nearest the fan wall under high ventilation, where the coefficient is about five times larger than any other group-bin combination; the response is not uniform across the house.

**Figure 5:**
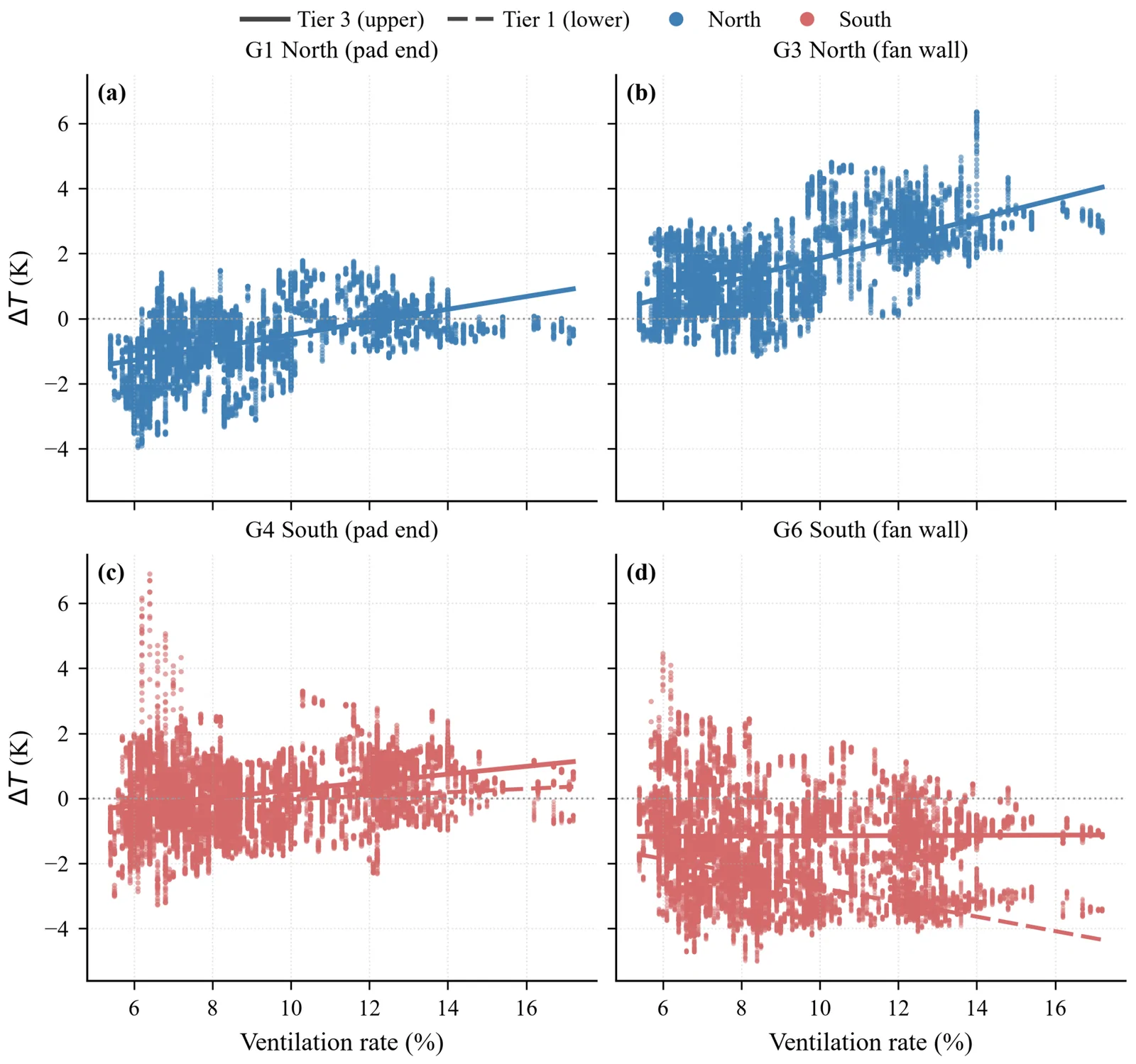
Continuous ventilation coupling: 1-min $\Delta T=T_{1.5m}-T_{9.5m}$ (K) versus ventilation rate (%). Panels: (**a**) G1, (**b**) G3 (north), (**c**) G4 and (**d**) G6 (south). Colour = sidewall (blue = north, red = south); larger/darker markers = Tier 3, smaller/lighter = Tier 1; solid lines = Tier 3 OLS fits, dashed = Tier 1 fits. The dashed fit appears in panels (**c**,**d**) only, because the two north-side 9.5 m Tier 1 sensors were excluded (Section 2.2). Panels share y-axis limits; slopes, *r* and *p* values are given in Table 3.

In the same framework, the ventilation rate was also treated as a continuous variable. The 1-min ΔT series was regressed on the ventilation rate (%) via ordinary least squares (OLS) for each group-tier combination (Figure 5; Table 3). On the north side, ΔT increased significantly with ventilation rate (G3 Tier 3: slope 0.303 K per %, r=0.62, $R^{2}=0.39$, p<0.001; G1 Tier 3: 0.196 K per %, r=0.51, $R^{2}=0.26$, p<0.001), consistent with higher ventilation strengthening the near-inlet gradient. The south side showed weaker (G4: 0.12 K per %, r=0.30) or non-significant coupling (G6 Tier 3: 0.004 K per %, p=0.37, r=0.01), in line with the spatial heterogeneity described above.

During the analysis window the controller’s ventilation position covered 5.2–17.2% of its range (median 8.0%), within the age-indexed minimum-ventilation band (Section 2.1). Ventilation was higher by day than at night (mean 9.7% vs. 8.0%), the side-window opening averaged 22.7% by day and 18.9% at night (range 10–51%), and the longitudinal inlets remained closed throughout (opening about 1%), so the side-wall inlets were the only supply path during the analysis window as well. Adding the recorded indoor humidity, negative pressure and building-level CO2 to the regressions leaves the two north-side slopes that carry the result within −18% to +36% across all covariate combinations, and negative pressure alone changes them by at most 3.1%; the near-zero south-side slopes are not stable to covariate choice; given the collinearity with ventilation rate (Section 2.5), these adjusted coefficients are reported as a sensitivity check rather than as causal adjustments.

### 3.3. Vertical Thermal Stratification

While the near-inlet temperature decay is near-instantaneous, sustained temperature differences between upper and lower cage tiers would expose birds at different heights to systematically different thermal conditions. The vertical temperature gradient and its dependence on side and ventilation were quantified next. Throughout this section ΔT denotes the vertical tier difference (Tier 3 minus Tier 1), which is distinct from the horizontal $\Delta T=T_{1.5m}-T_{9.5m}$ used in Section 3.1 and Section 3.2.

**Figure 6:**
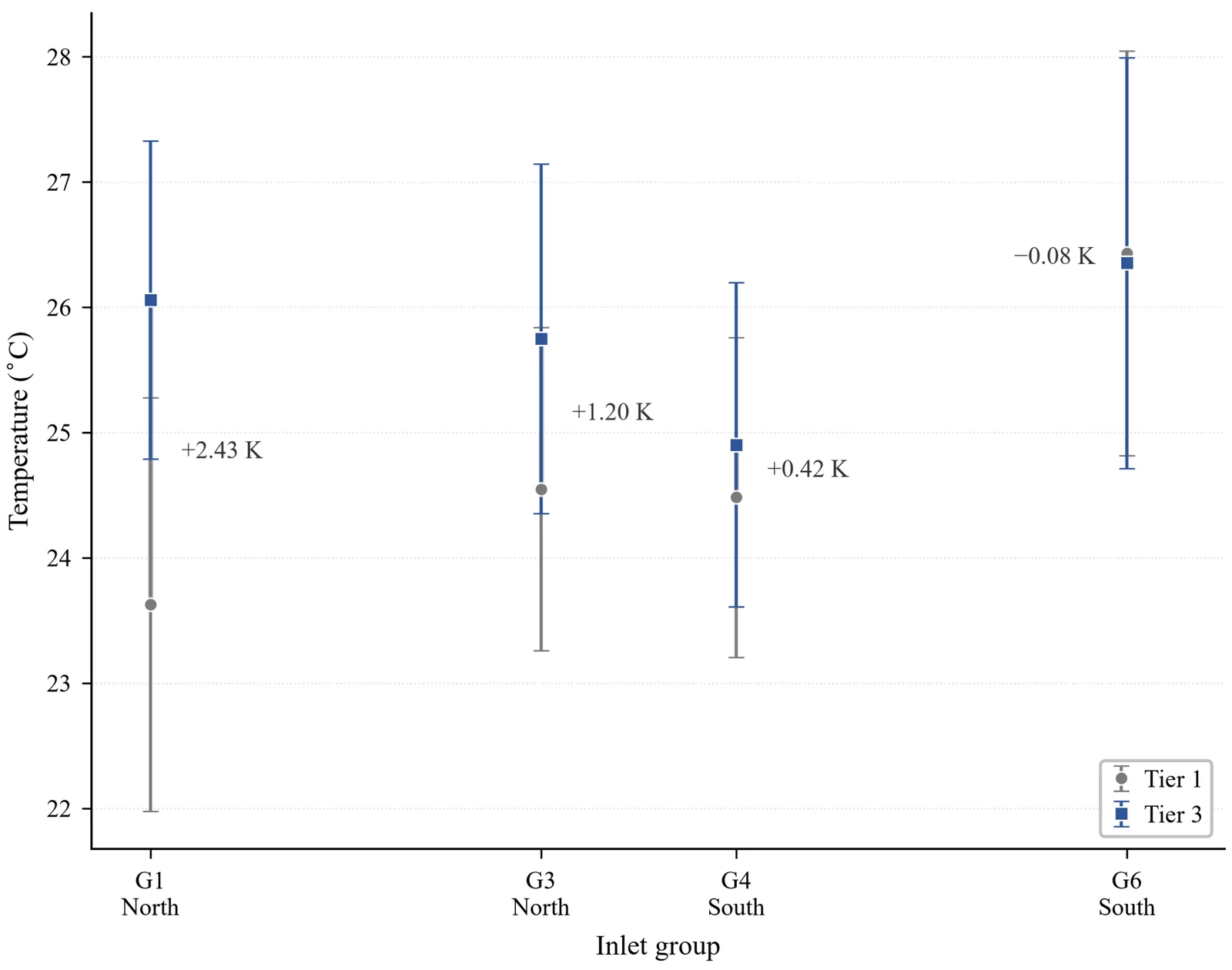
Vertical stratification by inlet group. Circles = Tier 1 (0.5 m), squares = Tier 3 (2.1 m); annotated values are ΔT (K), and error bars are ±1 SD. North-side Tier 1 statistics rest on the 1.5 and 5.5 m sensors only, because the two 9.5 m Tier 1 sensors were excluded (Section 2.2), so the north-side difference may be biased by the missing far-field points (Section 3.3). The Group 6 Tier 3 mean includes a degraded sensor retained where valid (Section 2.2); excluding that sensor changes the group’s tier difference by 0.21 K (Section 3.3).

Mean vertical stratification, pooling all valid records, was 0.78 K (Tier 3: 25.72 °C, Tier 1: 24.94 °C; Cohen’s d=0.47; Figure 6; Table 4). North-side Tier 1 means are computed from the 1.5 m and 5.5 m sensors only (9.5 m Tier 1 sensors 1-27-1/3-27-1 fully missing). The effect of restricting a tier to two distances can be gauged from the south-side groups, where all three distances were recorded: truncating to the two near distances changed the tier means by 0.002–0.017 K in Group 4, whose distance gradient is weak, and by 0.51–0.70 K in Group 6, whose gradient is large. The north-side groups bracket both behaviours, so the direction of the net effect of the missing points cannot be established from the available record; the north-side stratification is reported as a two-distance statistic and the missing Tier 1 cells are marked in Table 2.

The two degraded sensors (Section 2.2) do not drive these values. Excluding both entirely changes the group tier means by at most 0.21 K; the tier differences of Table 4 move from −0.08 to +0.13 K in Group 6 and from 0.42 to 0.46 K in Group 4, while the north-side values are unchanged, because the degraded sensors sit at 5.5 m and enter no $\Delta T=T_{1.5}-T_{9.5}$ pair. Group 6’s tiers therefore did not differ measurably, and the sign of that difference is not resolvable; both of its values are smaller than the sensors’ rated accuracy (±0.2 K).

Two-way ANOVA (Type II, Table 5) indicated significant effects of side (F=6099, p<0.001, $\eta_{p}^{2}=0.021$), tier (F=19,680, p<0.001, $\eta_{p}^{2}=0.064$), and their interaction (F=25,882, p<0.001, $\eta_{p}^{2}=0.082$). Residual diagnostics showed mild non-normality and heteroscedasticity (Shapiro-Wilk on 5000 sampled residuals: W=0.9962, p<0.001; Levene across 4 side × tier cells: F=2038.7, p<0.001), but with n=288,080 the ANOVA is robust; a linear mixed-effects model with a random intercept for sensor confirmed the same fixed-effect pattern (sensor variance 0.99, residual 1.67, intraclass correlation coefficient ICC =0.37, i.e., sensor position accounted for 37% of the total variance). The interaction reflects larger north-side differences (Group 1: 2.43 K) than south-side ones (Group 4: 0.42 K). On the south side, both groups showed small tier differences—Group 4 0.42 K and Group 6 none measurably—consistent with daytime radiative gain reducing stratification on the exposed side (Section 3.5).

### 3.4. Time-Varying Flow Regime and Buoyancy

**Figure 7:**
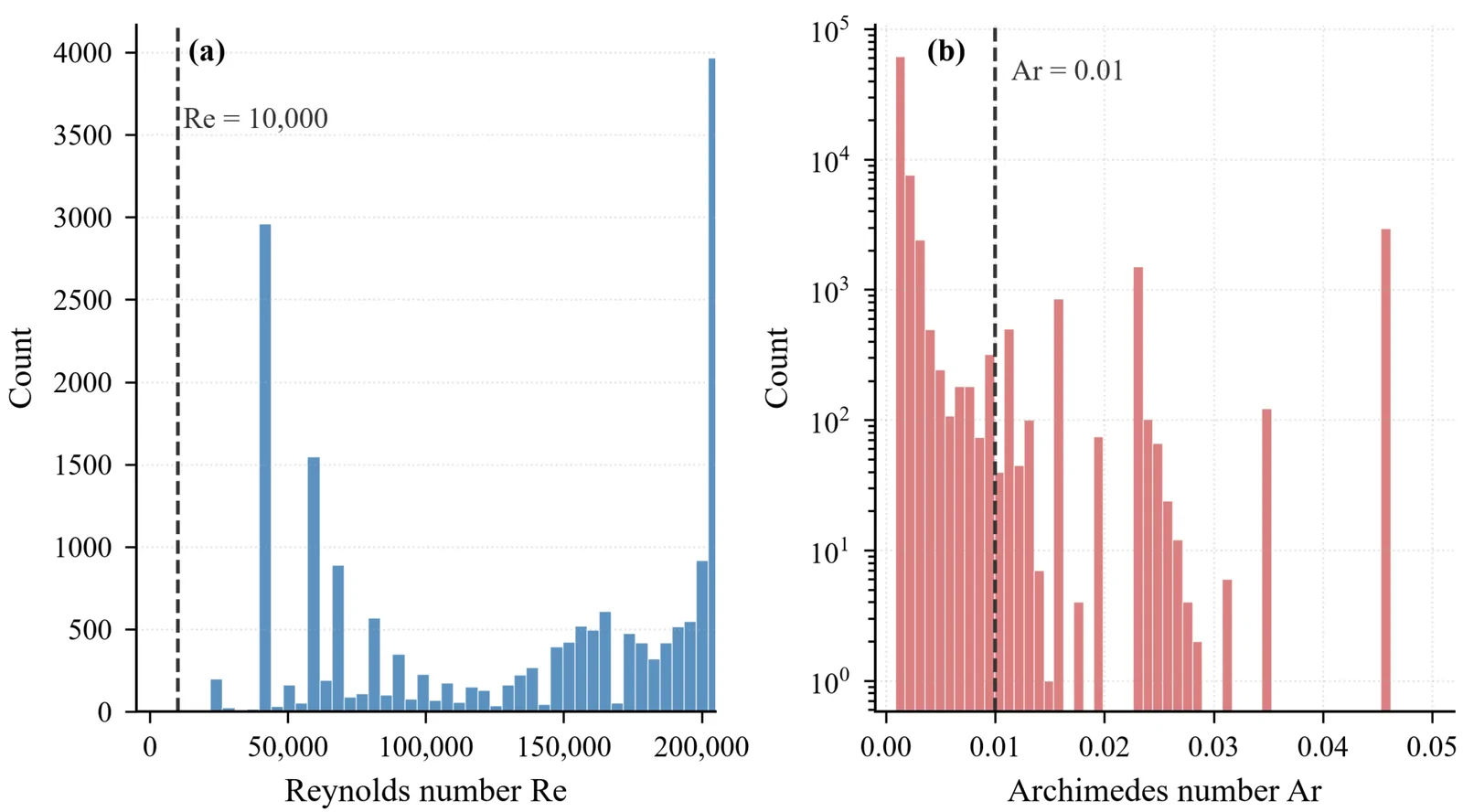
Re and Ar distributions (time-varying flow regime). (**a**) Re and (**b**) Ar distributions (active ventilation only); dashed lines mark Re=10,000 and Ar=0.01, and the axis in (**b**) is truncated at Ar=0.05. Only the north-side pressure positions were available (P1, P5, P9, P13), so no south-side Ar is shown.

The inlet velocity, Reynolds number, and Archimedes number all vary as the controller modulates fan operation. Using 1-min medians of the static-pressure record (ΔP range 0–51 Pa within the analysis window; the underlying 10-s record spans 0–57 Pa over 3.3 M values; filtered to Re <220,000 for display), the time-varying distributions were Remedian=203,000 (p5 3000, p95 269,000, corresponding to $v_{\mathrm{inlet}}\approx 6.5$ m/s) and ${\mathrm{Ar}}_{\mathrm{median}}=0.0018$ (Equations (4) and (5), with the representative 5 K jet–ambient difference), both well below the 0.01 buoyancy threshold of Leonard and McQuitty [23]. 57.2% of all records fell below Ar=0.01 (Figure 7), with the excess coming almost entirely from near-zero-pressure records when ventilation was idle. During active ventilation (ΔP>1 Pa), Ar was consistently low. The dimensionless decay coefficients $\alpha \cdot d_{h}$ ranged from 0 (Groups 1, 6) to 0.0018 (Group 3) for the window as a whole, and up to 0.004 in the most ventilated tertile—at least 2.5 times below the lower bound of the 0.01–0.03 range for free slot jets [5].

### 3.5. Spatiotemporal Temperature Field and Outdoor Coupling

**Figure 8:**
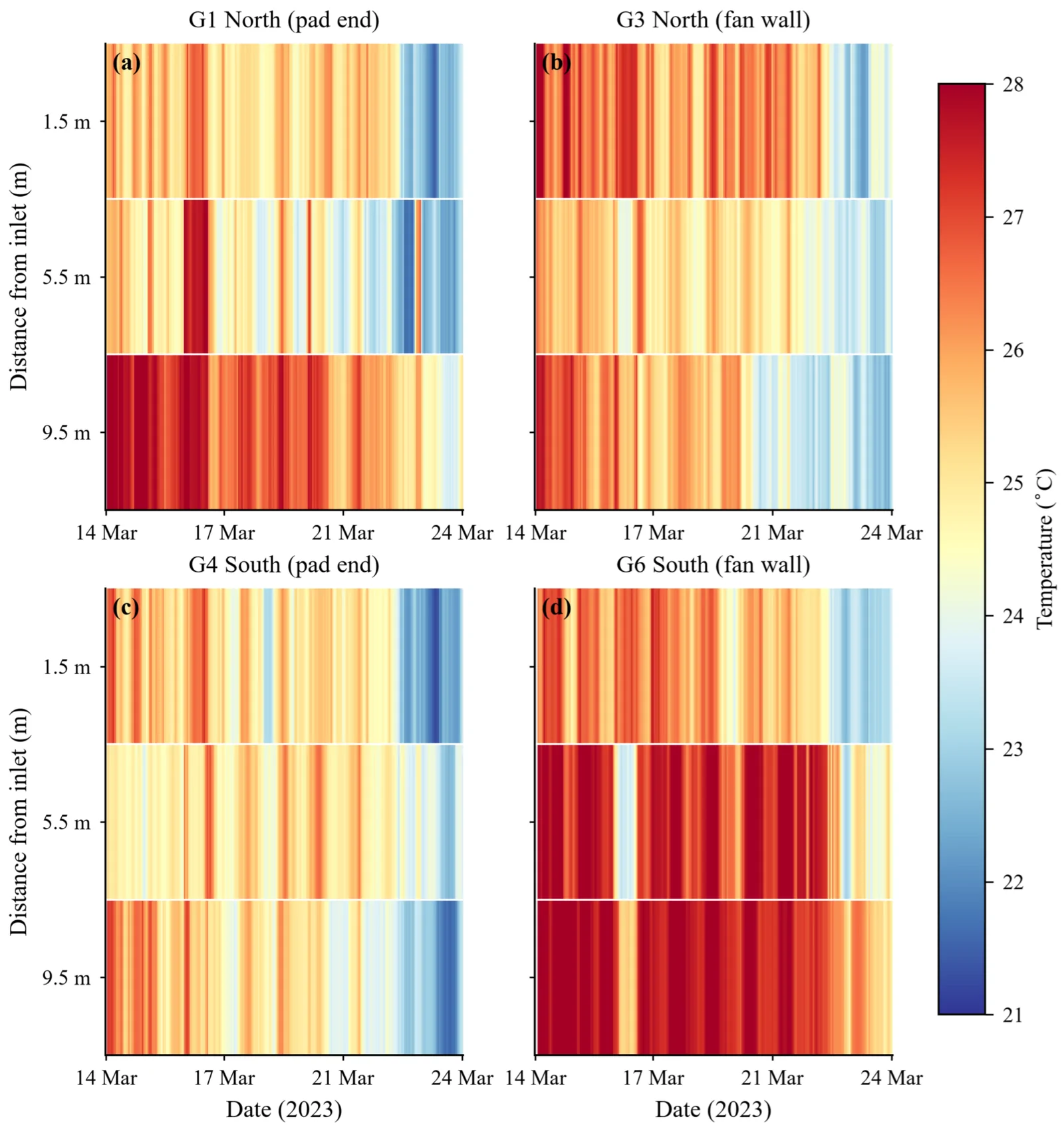
Spatiotemporal heatmaps of hourly mean temperature (1-min cage data aggregated to 1 h). Panels: (**a**) G1 and (**b**) G3 (north), (**c**) G4 and (**d**) G6 (south); colour = temperature (°C), y = distance from the inlet wall (1.5/5.5/9.5 m), x = date (14–24 March). Grey cells mark missing hourly means; the degraded sensor 6-17-3 is masked.

At hourly resolution, the spatiotemporal heatmaps (distance × time; Figure 8) show that the three measurement positions are separated by less than 1 K in about one fifth of the group-hours (median spread 1.8 K), and that the largest contrasts occur in Groups 1, 3 and 6. Across the measured span the group-mean ΔT reaches +1.56 K in Group 3 (Tier 3, d=1.26) and −2.53 K in Group 6 (Tier 1, d=−2.09), the latter opposite in sign to the near-inlet cooling expected from a cold supply jet. Groups 1 and 4 change by less than 0.7 K in the group mean (−0.69 K at Tier 3 in Group 1; −0.08 and +0.14 K in Group 4), although the hour-to-hour spread within Group 1 reaches a median of 2.3 K. The 1.5 m position is therefore not systematically the coldest one; the sign of the near-inlet contrast is group-specific.

Outdoor and ventilation coupling at the hourly scale (Spearman, Section 2.6; Table 6) showed that ventilation rate had the strongest monotonic association with the horizontal ΔT in the two north-side groups ($r_{s}=0.56$), followed by outdoor temperature; wind speed had little explanatory power. On the north side, ΔT correlated positively with both ventilation rate (G3: $r_{s}=0.56$, p<0.001; G1: 0.56, p<0.001) and outdoor temperature (G3: 0.51, p<0.001; G1: 0.52, p<0.001), while wind speed was weak or non-significant (G1: $r_{s}=0.06$, p=0.33). Group 3 also showed a day-night difference in the horizontal contrast (Tier 3, the only tier with a complete 1.5–9.5 m pair in that group; day +1.87 K vs. night +1.38 K, p<0.001; Table 6).

**Figure 9:**
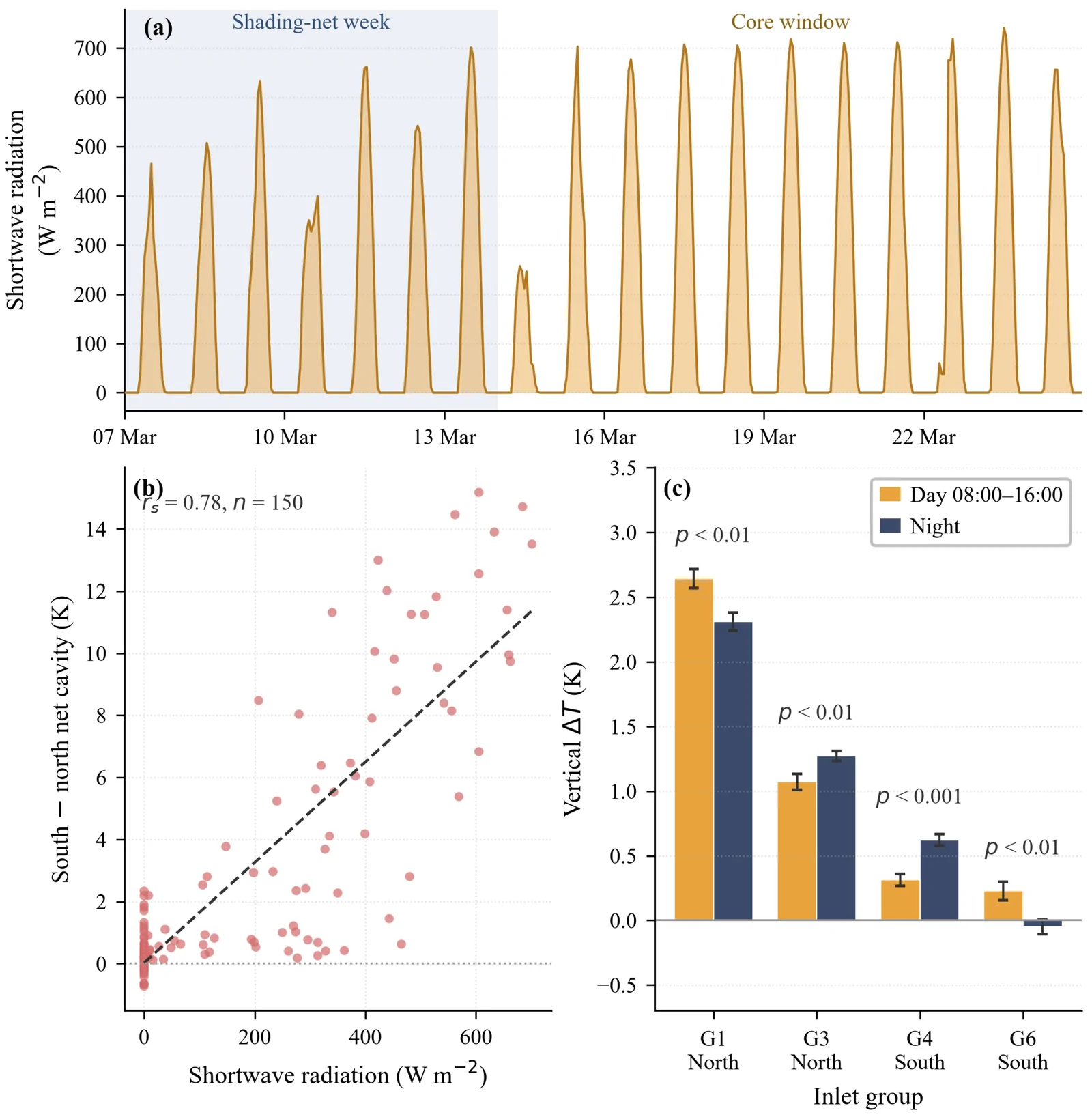
Reanalysis radiation and its coupling to the recorded contrasts. (**a**) Hourly shortwave radiation from ERA5 for the site over the two measurement windows (shading-net week 7–13 March; core window 14–24 March). (**b**) Shading-net week: hourly south-minus-north difference of the net-cavity air temperature against radiation ($r_{s}=0.78$, n=150). (**c**) Core window: vertical ΔT (Tier 3 − Tier 1) by group in daytime (08:00–16:00) and at night; bars are group means and error bars are ±1 standard error of the hourly means, with Mann-Whitney *p* values above each pair.

To test the radiative component directly, hourly shortwave radiation for the site was taken from the ERA5 reanalysis (Section 2.3). Daytime radiation averaged 350.7 W m^−2^ over the analysis window (maximum 741 W m^−2^), and radiation was only moderately collinear with the in-house variables (Spearman $r_{s}=0.39$ with ventilation rate and 0.44 with outdoor temperature), so it adds information beyond them. The horizontal near-inlet contrast was not primarily radiative: its correlation with radiation was weak in every group ($r_{s}=0.10$, 0.21, −0.02 and −0.14 for G1, G3, G4 and G6; Table 6), and the two nominally significant cases had opposite signs. The vertical contrast instead carried a day-night structure consistent with radiative gain (Figure 9c): on the south side, Group 4 was about half as stratified by day as at night (0.31 vs. 0.62 K, p<0.001), whereas Group 6’s vertical difference stayed small at both times of day (day +0.23 K vs. night −0.05 K), although the day-night contrast is statistically detectable (p=0.003); the sign of that difference is not resolvable (Section 3.3). Within daytime hours alone, radiation added no further monotone signal to any contrast ($|r_{s}|\leq 0.20$, all p≥0.07). Group 6’s inverse horizontal contrast at Tier 1 (−2.53 K overall) was stronger by day than at night (day −2.81 K vs. night −2.37 K, p<0.001), so it carries a daytime component, yet within daytime hours it was only weakly related to irradiance; it remains an unexplained group-level feature of this house.

The outdoor temperature course is a pronounced diurnal cycle (range −10.1 to 16.3 °C, median 0.4 °C, with 46% of records below 0 °C), whereas the four near-inlet indoor series remained within a narrow band (21.2–28.2 °C) and tracked one another closely; the between-group differences were therefore small compared with the diurnal outdoor swing.

### 3.6. Shading Net Effect

Shading nets are routinely installed to block wind at the inlet, but their thermal effect depends on the balance between wind shielding and solar heating—a trade-off that differs between north- and south-facing sides in winter. The net thermal impact was evaluated with paired inlet/net sensors.

**Figure 10:**
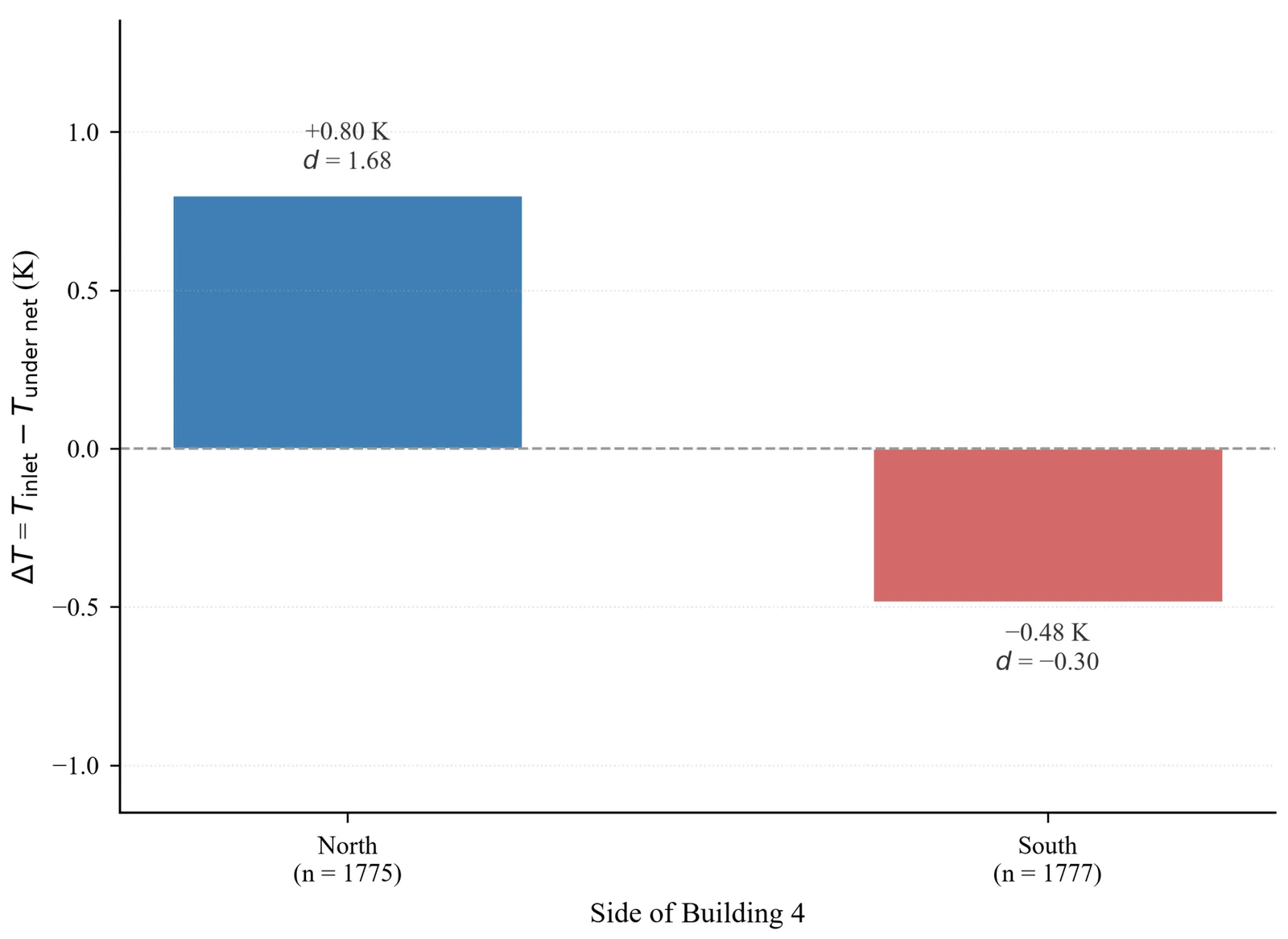
Shading net effect on inlet temperature. Blue = north side, red = south side; positive ΔT (K) indicates that the net lowered the temperature.

North-side shading nets reduced inlet temperature by 0.80 K (t=70.9, Cohen’s d=1.68, p<0.001; Figure 10; Table 7). On the south side, the net produced a smaller, reversed effect (ΔT=−0.48 K, d=−0.30, p<0.001), with shaded sensors recording slightly higher temperatures than unshaded ones. This paired comparison uses 5-min records from 7–13 March. The same week’s reanalysis radiation makes the mechanism measurable: the south-side net effect (inlet minus under-net temperature) scales with radiation ($r_{s}=-0.75$ over all hours and −0.74 within daytime, n=150 and 60 hourly means), while the north-side effect is weaker and positive over all hours ($r_{s}=0.32$, p<0.001; not significant within daytime, p=0.07); the south-minus-north difference of the net-cavity temperature, which expresses the side asymmetry of the trapped air, correlates strongly with radiation ($r_{s}=0.78$, p<0.001; Figure 9b). A cross-side contrast of this kind cannot by itself separate the net’s effect from the two sides’ different incident radiation; what the radiation scaling adds is that the reversal tracks the radiative input. The paired loggers also record the air temperature under each net. Over the daytime window (08:00–16:00, n=695 paired 5-min records), the south-side net cavity averaged 9.68 °C against 3.62 °C on the north side, a mean south-minus-north difference of 6.06 K (median 5.56 K), so the south-side net cavity was the warmer of the two over the same window.

The shading-net comparison was run as a separate week-long paired experiment and is not used in the core decay analysis of Section 3.1, Section 3.2, Section 3.3, Section 3.4 and Section 3.5. The two windows differ in flock age (days 21–27 vs. 28–38) and weather (mean outdoor temperature 0.50 vs. 1.80 °C; mean wind speed 2.9 vs. 4.6 m s^−1^; mean ventilation position 5.1% vs. 8.6%). The paired design removes these differences for the within-window net-minus-inlet contrast, because both members of each pair were logged at the same time and place; what the window difference bounds is the magnitude of the effect, which the radiation scaling shows to be weather-dependent, not its side-dependent sign.

## 4. Discussion

The discussion follows the results in order. Section 4.1 addresses the near-inlet gradient and its dimensionless interpretation (Section 3.1 and Section 3.4), Section 4.2 the ventilation dependence (Section 3.2), Section 4.3 the vertical stratification and its coupling to outdoor conditions (Section 3.3 and Section 3.5), Section 4.4 the shading nets (Section 3.6), Section 4.5 the welfare implications of the measured heterogeneity, and Section 4.6 the limitations.

### 4.1. Why No Near-Inlet Gradient Is Resolved in the Bird Zone

With only three measurement distances per group, the exponential decay model could not reliably resolve decay for three of four groups (standard errors comparable to the estimates). The complementary ΔT analysis, which uses the full time series (n>14,000 per group per tier), resolves the picture more finely but not into a single pattern. Group 4 changed by less than 0.15 K across the domain, Group 1 by −0.69 K at Tier 3, Group 3 by +1.56 K (d=1.26), and Group 6 by −2.53 K at Tier 1 (d=−2.09). Two features of that list matter. Only Group 3 has the sign a cold supply jet must produce; Group 6 has the largest change of all and the opposite sign, so its behaviour cannot be a jet signature, and the same conclusion follows from the dimensionless comparison, where the measured $\alpha \cdot d_{h}$ (0 to 0.0018) is a factor of five or more below the lower bound of the 0.01–0.03 design range for slot jets [5,6]. Whether the near-inlet profile is resolved depends on spatial resolution rather than sampling rate: the mixing time scale ($t_{\mathrm{mix}}\approx 0.5$ s) is negligible against the 1-min interval, but the first sensor sits 1.5 m from the wall, so the analysis captures the net change across the measured domain, not the shape of the profile within it.

The same reasoning explains why vertical stratification, measured over the 1.6 m span between the two instrumented tiers, emerges as the dominant inhomogeneity while the near-inlet decay does not. Cages, birds and equipment are expected to obstruct the jet and to drive mixing into a regime outside the free-jet correlations, although no velocity measurement was available to verify this directly (Section 4.6). This is consistent with Hoff and Wu [20], who found installed inlet performance departing from laboratory predictions, with Chu et al. [36] on flap configuration, and with CFD studies of confined livestock buildings in which internal obstructions dominate over free-jet mechanics [25,37]. The finding is therefore not that mixing is absent but that a cold-jet cooling gradient no longer survives to the nearest instrumented position (1.5 m) in three of the four groups, and where it does, the coefficient is far below the free-jet design range; for the bird-occupied zone the free-jet coefficient is therefore at best a weak descriptor, not the design variable.

What the thermal record can say about the flow path is indirect. The absence of a resolvable near-inlet gradient at 1.5 m, together with the strong vertical contrast, is consistent with a jet that is deflected and broken up by the cage array. Cold air entering at 3.4 m height either mixes with the upper-zone air above the cages or descends through the cage decks; by the first measurement plane, the horizontal signature of the jet has been erased while the vertical signature (cooler lower zones, warmer upper zones) persists. Published CFD of cage and floor houses shows the same qualitative behaviour—internal obstructions and the bird-occupied zone dominate the jet trajectory, and the resulting airflow departs strongly from free-jet predictions [12,24,38]—and recent three-dimensional simulations that represent the cage zone as a porous medium reach the same conclusion [39]. Those studies also show, however, that a trajectory cannot be inferred from temperature alone. Air-velocity, tracer and visualisation measurements were not available here, so the flow path remains inferred rather than observed.

These results complement the house-level assessment of Deng et al. [29] conducted at the same farm. That study characterized longitudinal and lateral temperature gradients across the entire building during the same winter period, identifying solar radiation and wind as primary drivers of lateral non-uniformity.

The present work extends those findings by characterizing the near-inlet thermal field between 1.5 and 9.5 m from the sidewall—a scale that Deng et al. [29] did not address—and by providing the vertical (inter-tier) detail that zone-specific control would need.

### 4.2. Ventilation Dependence of the Decay

Measurable decay emerged under high ventilation, and substantially only in Group 3 (α = 0.0084 m^−1^; Group 4 reached 0.0015 m^−1^, five times lower), so the phenomenon is condition-dependent rather than uniformly absent. Higher ventilation raises inlet velocity and jet momentum and lengthens the penetration zone, broadly consistent with the jet-drop models of Zhang et al. [18] and Zhang and Strøm [19] and with the direction of $\alpha \propto 1/v_{\mathrm{inlet}}$; the relationship remains correlative here, and quantitative validation would require direct velocity measurement at each group. The three-point fit also under-estimates the magnitude, predicting a 0.75 K difference for Group 3 where the time series gives 1.56 K. What remains unexplained is why Group 3 alone responds: it sits near the exhaust-fan wall, where local negative pressure is highest, whereas Group 1 at the opposite end and Group 4 on the sun-exposed south side stayed near zero even at high ventilation.

Differences in local static pressure, inlet flap angle, or upstream obstructions are the plausible candidates—Cheng et al. [38] showed that such factors substantially alter simulated airflow distribution, and the turbulence-model choice affects those predictions in turn [40]. Wheeler et al. [41] found that temperature and velocity uniformity in tunnel-ventilated broiler houses depends strongly on inlet configuration, Hu et al. [42] built a ventilation-optimization framework that explicitly accounts for spatial heterogeneity, and Saner and Shekhawat [43] argued for climate-responsive inlet design. The practical reading is that inlet response varies by position within a single house: only the group nearest the exhaust-fan wall developed a resolvable decay even at high ventilation, the north and south sides responded differently at the same ventilation rate, and sensor position accounted for 37% of the variance in the mixed-effects model (Section 3.3). This argues for monitoring and control resolved by tier and by side rather than a single house mean (cf. Luo et al., 2026 [7]), and predictive control approaches that follow the flock’s changing thermal demand [8] would similarly benefit from position-resolved sensing; a staged zone-by-zone control scheme is a plausible extension of this result, but it was not tested in this campaign.

### 4.3. Vertical Stratification in Context: Magnitude and Tolerance

The 0.78 K mean difference between Tier 3 and Tier 1 sits at the lower end of the 1–3 K range reported by Wheeler et al. [10] for floor-to-ceiling gradients. The smaller magnitude here probably reflects the shorter vertical span between measurement planes (1.6 m rather than the full building height) and the cage decks that impede vertical free convection, and it is consistent with the 0.5–2.0 K range of Boshouwers et al. [11].

What matters practically is the spread between groups rather than the mean: north-side stratification reached 2.43 K in Group 1, exceeding the ±1–2 K tolerance within which broiler houses are normally operated [44] and which Saeed et al. [45] and Chen et al. [2] identify as influential for bird health and productivity. The side × tier interaction ($\eta_{p}^{2}=0.082$) shows that the gradient is not uniform. On the north side the lower tier was the coldest zone, consistent with cold incoming air accumulating near the floor and with the absence of solar gain. On the south side the tier differences were small—Group 4 at 0.42 K and Group 6 with no measurable difference (Section 3.3)—and Group 4 was about half as stratified by day as at night (Section 3.5), the direction expected if daytime radiative gain warms the exposed side. Deng et al. [29] reported a strong correlation between solar radiation and the north–south temperature difference at this facility (Spearman R = 0.610, *p* < 0.001), and the present reanalysis-based test reproduces that side asymmetry where it can be measured directly, in the shading-net week (Section 4.4). The partitioning between inlet-driven cooling and envelope heat gain still cannot be resolved from temperature data alone at the tier level, and no airflow measurement is available to attribute either term to a specific jet trajectory. Shah et al. [46] showed that stratification can be reduced through targeted ventilation during brooding and cool weather, which is the direction these results support. The practical finding is that birds on different tiers, and on different sides of the same house, experience systematically different conditions, and current ventilation control does not address it.

### 4.4. Shading Nets: Side-Dependent Effects

The north-side shading net lowered inlet temperature by 0.80 K with a large effect size (d=1.68). For a house drawing 3280–60,020 m^3^ h^−1^ of outdoor air near freezing (the design minimum-ventilation range for this house), this reduction translates to a non-trivial increase in ventilation heat load (colder inlet air requires more heating to maintain house temperature). The south-side reversal (ΔT=−0.48 K) tracks the radiation record within the same week: the south-side net effect scales with shortwave radiation ($r_{s}=-0.75$), and the south-minus-north difference of the net-cavity temperature correlates at $r_{s}=0.78$ (Section 3.6). This is consistent with the net trapping solar-heated air near the inlet on the exposed side, offsetting the wind-blocking benefit. Over the daytime window the air under the south-side net averaged 9.68 °C against 3.62 °C under the north-side net, a difference of 6.06 K (Section 3.6), indicating that the south-side net cavity is the warmer of the two in daytime.

This is a within-building paired comparison, so between-building differences cannot confound the sign reversal. The two windows differ in flock age and weather (Section 3.6), which bounds the magnitude of the effect rather than its side-dependent sign: the comparison is self-contained, based on simultaneous pairs, and is not used in the core decay analysis, and the radiation scaling measured here explains why the magnitude should not be transferred to other weather conditions. Deng et al. [29] reported a strong positive correlation between outdoor solar radiation and the south–north difference in net-cavity temperature (Spearman R = 0.709, *p* < 0.001) at the same facility, consistent with the $r_{s}=0.78$ obtained here from simultaneous in-net pairs. More broadly, Kuczynski et al. [47] showed that window shading and thermal mass interact to determine building thermal comfort. By analogy, shading modifies the radiation balance in agricultural structures: roof whitening (whitewash) alters greenhouse microclimate [48], and shade screens change both microclimate and crop transpiration in protected cultivation [49]. The same physical principle—reduced radiative gain and altered convective exchange—applies to the shading nets used on livestock housing, although here the nets are installed to block wind rather than to control radiation. In this house, selective or adjustable shading would be the natural response—beneficial on north-facing inlets but potentially counterproductive on south-facing ones during sunny winter days.

### 4.5. Welfare Implications and Precision Monitoring

Placed against welfare criteria, the measured heterogeneity is not only a control problem. The tier contrast reached 2.43 K in Group 1, and the means across the eight instrumented group-tier zones spanned about 2.8 K (23.6 to 26.4 °C), while broiler houses are normally operated within a ±1–2 K band [44] and cold stress and heat stress are recognised welfare consequences for broilers [30]. Birds housed at different tiers, and on different sides of the same house, therefore experienced systematically different thermal conditions at the same time, and the controller, which regulates a single house-average temperature, cannot distinguish them. The logger accuracy (±0.2 K) is small against the tier contrasts that were resolvable (0.42 to 2.43 K), so these differences are real at bird level rather than sensor noise.

The monitoring approach used here, continuous in-cage records resolved by tier and side, is the resolution that welfare-oriented assessment needs. Automated, continuous monitoring of behaviour and environment is the core of precision livestock farming for poultry welfare [32], and such technologies are promoted as a way to make welfare assessment continuous rather than episodic [33]. Thermal maps of the kind shown in Figure 8 fit this framework directly, because they can be produced from the sensing layer that already serves ventilation control; what they add is the ability to see, in the controller’s own record, which groups of birds sit outside the intended band and for how long.

Practically, the results argue for management resolved by tier and side rather than by the house mean: monitoring and alarm thresholds set per tier and per side, shading evaluated separately for the two sides because its thermal effect changes sign between them, and inlet adjustments checked against in-cage records rather than against design coefficients alone. Two limits bound these implications. First, no animal-based welfare indicators were measured in this campaign: no behaviour, activity, feed intake, body weight or mortality records were kept, so the welfare reading is inferential, resting on established thermal-comfort ranges rather than on observed bird responses. Second, the evidence covers one house and one winter window under minimum ventilation. Pairing tier-resolved thermal monitoring with behavioural or physiological welfare indicators is the natural next step, and it is the step that would turn the spatial resolution demonstrated here into a welfare assessment in its own right.

### 4.6. Limitations and Future Work

Several limitations bound these results. The exponential decay model rests on only three distances per group (n=3), so the analysis captures the aggregate change across the measured domain rather than the continuous trajectory of jet decay, the precision of α is low, and the candidate model forms cannot be separated; physical interpretation rests on the complementary ΔT analysis, which is statistically robust but does not resolve the shape of the decay profile. Only two of four tiers were instrumented (Tier 1 at 0.5 m and Tier 3 at 2.1 m, a 1.6 m span), so intermediate stratification must be inferred. Static pressure—and therefore Re(t) and Ar(t)—was available only on the north side (positions P1, P5, P9, P13), so the physical model $\alpha \propto 1/v_{\mathrm{inlet}}$ (Equation (3)) rests on north-side data alone; a 3.4-day record of the south-side damper pressure in the same house shows a south-minus-north difference with a median of +5 Pa and 5th–95th percentiles of −27 to +33 Pa, with 24% of samples differing by more than 20 Pa. Side-to-side differences of the same order as the driving pressure therefore do occur, and the north-side values should be read as representative of one side. The core analyses cover a single 10-day winter window (14–24 March, bird age 28–38 days) in one house under minimum ventilation, and the shading-net comparison comes from a separate, adjacent week (7–13 March); the campaign covered the final stage of one batch, so seasonal variation, house-to-house differences and between-cycle procedures are not captured.

Temperature alone cannot characterise jet trajectories, recirculation or local air movement, and no airflow visualisation (smoke or PIV), anemometry or tracer measurement was available at the inlet groups, so inferences about jet penetration, mixing distance and the role of cage-tier obstructions rest on the thermal signature combined with pressure-derived velocity and dimensionless criteria (Section 4.1). No laboratory experiments or computational fluid dynamics simulations were performed in this campaign, so the comparison with the published simulations cited in Section 4.1 is qualitative. Air velocity within the cages, in-cage CO_2_ and dust were not measured in this house, and recent surveys show that bioaerosol and gas loads in winter cage houses vary strongly with bird age and position [50,51]; combining such measurements with the temperature field would connect the air-quality and thermal pictures. Humidity, CO_2_ and NH_3_ were not mapped at the inlet groups (CO_2_ was available only as the controller’s building-level channel; Section 2.3). Radiative effects are represented by hourly ERA5 reanalysis radiation for the site rather than by an on-site pyranometer. The reanalysis supplies the day-to-day and daytime course used here, but it cannot be checked against on-site radiation in this campaign. It also does not resolve local shading or the building’s own radiation field, so the radiative tests are a regional-scale check rather than a local measurement. Feed was delivered three times daily with ad libitum access, and the window spanned a scheduled lighting change at about day 31/32, so the flock experienced both the dimmed and the full-illumination programme; no continuous feed-intake or activity logging was available to enter metabolic heat production as a covariate.

Repeating the key contrasts on heater-off records and on the two halves of the window stabilises these management concerns. The heating circuit switched on intermittently (about 13% of controller records, concentrated on 14–16 March); excluding those records changed group means by at most 0.11 K and tier differences by at most 0.06 K. The split-window reruns preserved the sign and rank order of the principal contrasts; the only sign change was Group 6’s near-zero tier difference (+0.31 K before vs. −0.07 K after the lighting change, i.e., near zero in both halves). Resolving the decay profile directly, and separating management-driven from ventilation-driven signals, would require 5–8 measurement points within the first 5 m, synchronized activity and lighting logs, and paired anemometry or particle image velocimetry [52]; multi-criteria evaluation frameworks [53], digital-twin monitoring [54], CFD validation practice [55] and IoT-based sensing [56] are the relevant methodological directions.

## 5. Conclusions

In a densely caged multi-tier broiler house under winter minimum ventilation, the bird-level thermal environment was organised by cage tier and side of the house rather than by distance from the side-wall inlets. Three of the four instrumented groups showed no resolvable cooling gradient at the nearest measurement plane, 1.5 m from the inlet wall; the fourth group (north side, nearest the exhaust-fan wall) was the only one with a resolvable coefficient in its full-record fit, and even at high ventilation that coefficient remained at least 2.5 times below the free-jet design range ($\alpha d_{h}\leq 0.004$). The largest single contrast (about 2.5 K, south side near the exhaust-fan wall) had the opposite sign to a cold supply jet and is not explained by jet decay. Vertical stratification between the lower and upper instrumented tiers was the largest and most persistent source of inhomogeneity (0.78 K on average; up to 2.4 K in the north-side group at the pad end), and the two sides of the house responded in opposite ways to the same shading nets.

Mean temperatures across the eight instrumented group-tier zones spanned about 2.8 K (23.6 to 26.4 °C), exceeding the ±1–2 K band within which broiler houses are normally operated, while the controller regulates to a single house-average temperature. Monitoring and control resolved by tier and side would represent the conditions the birds actually experience, although the control benefit itself was not tested here; the same resolution shows which groups of birds fall outside the band within which the house is operated, which is the information a welfare-oriented assessment would need, although no welfare outcomes were measured here. Inlet designs carried over from free-jet correlations should be checked against in-cage measurements before they are trusted, and shading for wind protection should be evaluated separately for north- and south-facing inlets because its thermal effect changes sign between the two sides. The evidence covers one house and one winter window under minimum ventilation, and the transferable result is the structure of the finding—tier and side first, jet penetration second—rather than the coefficients themselves.

## Figures and Tables

**Table 1:** Exponential decay coefficients by inlet group, fitted over the full analysis window (α≥0 constraint).

Group	Side	T_0_ (°C)	α (m^−1^)	SE (α)	R^2^	$\alpha \cdot d_{h}$
1	North	25.33	0.0000	0.010	0.00	0.0000
3	North	25.75	0.0037	0.003	0.60	0.0018
4	South	24.75	0.0002	0.001	0.05	0.0001
6	South	26.41	0.0000	0.009	−0.00	0.0000

Note. α = decay coefficient; SE = standard error; $d_{h}$ = hydraulic diameter (0.47 m). α=0 indicates that the exponential model could not resolve decay within the measurement domain, not that the near-inlet contrast is absent: in one group that contrast was opposite in sign (Section 3.1). See ΔT analysis in the main text for a complementary, higher-resolution assessment.

**Table 2:** Mean temperature recorded at each instrumented position (14–24 March 2023).

Group	Side	Distance (m)	Tier 1 (°C)	Tier 3 (°C)	ΔT (K)
1	North	1.5	24.02 ± 1.37	25.85 ± 1.21	+1.83
1	North	5.5	23.23 ± 1.81	25.80 ± 1.21	+2.57
1	North	9.5	–	26.53 ± 1.26	–
3	North	1.5	24.97 ± 1.39	26.59 ± 1.33	+1.62
3	North	5.5	24.13 ± 1.02	25.62 ± 0.91	+1.49
3	North	9.5	–	25.04 ± 1.42	–
4	South	1.5	24.41 ± 1.36	25.01 ± 1.51	+0.60
4	South	5.5	24.75 ± 0.53 ^a^	24.83 ± 0.86	+0.08
4	South	9.5	24.49 ± 1.33	24.87 ± 1.41	+0.38
6	South	1.5	24.93 ± 1.17	25.99 ± 1.58	+1.06
6	South	5.5	26.92 ± 1.29	23.03 ± 0.69 ^b^	−3.89
6	South	9.5	27.46 ± 1.11	27.14 ± 1.01	−0.32

Note. Means ± 1 SD of the 1-min records; ΔT = Tier 3 − Tier 1 at the same position, in kelvin. ^a^ Sensor 4-17-1 contributed records for 28% of the window; ^b^ sensor 6-17-3 contributed records for 12% of the window and did not track its neighbouring sensor. Both cells rest on degraded records (Section 2.2) and are not used in any conclusion; the ΔT values in those two rows follow from the same records, and the sensitivity analysis in Section 3.3 removes both sensors entirely. Tier 1 at 9.5 m on the north side has no sensor (1-27-1 and 3-27-1 excluded).

**Table 3:** Continuous ventilation regression: ΔT (K) versus ventilation rate (%).

Group	Side	Tier	*n*	Slope (K per %)	SE	*p*	$R^{2}$	*r*
1	North	3	14,108	0.196	0.003	<0.001	0.26	0.51
3	North	3	14,120	0.303	0.003	<0.001	0.39	0.62
4	South	1	14,117	0.054	0.003	<0.001	0.03	0.17
4	South	3	14,122	0.121	0.003	<0.001	0.09	0.30
6	South	1	14,074	−0.222	0.004	<0.001	0.21	−0.46
6	South	3	14,075	0.004	0.004	0.37	0.00	0.01

Note. G1/G3 Tier 1 absent due to 100% missing in sensors 1-27-1 and 3-27-1 (14–24 March). G6 Tier 1 shows an inverse relationship that the reanalysis radiation record does not fully explain (Section 3.5), and it remains a group-specific feature of this house.

**Table 4:** Tier statistics by inlet group, pooled over the three measurement distances. Means in °C with ±1 SD, the SD being numerically identical in K; ΔT in kelvin (K), computed as Tier 3 − Tier 1 from the pooled position means. The sensitivity to the two degraded sensors is given in the text.

Group	Side	Tier 1 (°C)	Tier 3 (°C)	ΔT (K)
1	North	23.63 ± 1.65	26.06 ± 1.27	2.43
3	North	24.55 ± 1.29	25.75 ± 1.40	1.20
4	South	24.48 ± 1.28	24.90 ± 1.29	0.42
6	South	26.43 ± 1.61	26.35 ± 1.64	−0.08

**Table 5:** Two-way ANOVA (Type II): temperature versus side × tier.

Source	SS	df	MS	*F*	*p*	$\eta^{2}$	$\eta_{p}^{2}$
Side	15,019	1	15,019	6099	<0.001	0.018	0.021
Tier	48,461	1	48,461	19,680	<0.001	0.058	0.064
Side × Tier	63,732	1	63,732	25,882	<0.001	0.076	0.082
Residual	709,364	288,076	2.46	–	–	0.848	–

Note. n=288,080 cage-temperature observations (14–24 March). Mixed-effects sensitivity analysis (random intercept for sensor, n=80,000 random subsample): sensor variance 0.99, residual 1.67, ICC =0.37; fixed effects retained significance and direction.

**Table 6:** Hourly coupling of the horizontal ΔT ($T_{1.5m}-T_{9.5m}$, K) with outdoor and ventilation variables.

Group	Variable	*n* (hours)	$r_{s}$	*p*
1 (North)	Outdoor temp.	236	0.52	<0.001
1 (North)	Ventilation rate	236	0.56	<0.001
1 (North)	Wind speed	236	0.06	0.33
1 (North)	Shortwave radiation	236	0.10	0.12
3 (North)	Outdoor temp.	236	0.51	<0.001
3 (North)	Ventilation rate	236	0.56	<0.001
3 (North)	Wind speed	236	0.21	0.001
3 (North)	Shortwave radiation	236	0.21	0.001
4 (South)	Ventilation rate	236	0.26	<0.001
4 (South)	Shortwave radiation	236	−0.02	0.72
6 (South)	Ventilation rate	235	−0.28	<0.001
6 (South)	Shortwave radiation	235	−0.14	0.036

Note. Spearman correlations at hourly resolution (14–24 March). Outdoor data paired within 30 min, ventilation within 10 min; shortwave radiation from the ERA5 reanalysis (Section 2.3). Day (08:00–16:00) vs. night difference of the horizontal ΔT: significant in G3 only (its Tier 3, the only tier with a complete 1.5–9.5 m pair; day +1.87 K vs. night +1.38 K, p<0.001); the day-night structure of the vertical contrast is reported in Section 3.5.

**Table 7:** Shading net paired *t*-test results. ΔT in kelvin (K).

Comparison	*n*	ΔT (K)	SD (K)	*t*	*p*	Cohen’s *d*
B4 North: Inlet vs. Net	1775	0.80	0.48	70.9	<0.001	1.68
B4 South: Inlet vs. Net	1777	−0.48	1.60	−12.8	<0.001	−0.30

Note. Positive ΔT (K) indicates the shading structure reduced temperature. Paired 5-min records, 7–13 March 2023; the core analyses in Section 3.1, Section 3.2, Section 3.3, Section 3.4 and Section 3.5 use 14–24 March.

## Data Availability

The data presented in this study are available from the corresponding author upon reasonable request.
